# Multi-parametric cardiovascular magnetic resonance with regadenoson stress perfusion is safe following pediatric heart transplantation and identifies history of rejection and cardiac allograft vasculopathy

**DOI:** 10.1186/s12968-021-00803-7

**Published:** 2021-11-22

**Authors:** Nazia Husain, Kae Watanabe, Haben Berhane, Aditi Gupta, Michael Markl, Cynthia K. Rigsby, Joshua D. Robinson

**Affiliations:** 1 Department of Cardiology, Ann and Robert H. Lurie Children’s Hospital of Chicago, Chicago, USA; 2 Department of Pediatrics, Feinberg School of Medicine, Northwestern University, Chicago, USA; 3 Department of Biomedical Engineering, McCormick School of Engineering, Northwestern University, Chicago, USA; 4 Lincoln Medical and Mental Health Center, Bronx, NY USA; 5 Department of Radiology, Feinberg School of Medicine, Northwestern University, Chicago, USA; 6 Department of Medical Imaging, Ann and Robert H. Lurie Children’s Hospital of Chicago, Chicago, USA

**Keywords:** Pediatric heart transplantation, Cardiovascular magnetic resonance, CMR stress perfusion, Parametric mapping, Cardiac allograft vasculopathy

## Abstract

**Background:**

The progressive risk of graft failure in pediatric heart transplantation (PHT) necessitates close surveillance for rejection and coronary allograft vasculopathy (CAV). The current gold standard of surveillance via invasive coronary angiography is costly, imperfect and associated with complications. Our goal was to assess the safety and feasibility of a comprehensive multi-parametric CMR protocol with regadenoson stress perfusion in PHT and evaluate for associations with clinical history of rejection and CAV.

**Methods:**

We performed a retrospective review of 26 PHT recipients who underwent stress CMR with tissue characterization and compared with 18 age-matched healthy controls. CMR protocol included myocardial T2, T1 and extracellular volume (ECV) mapping, late gadolinium enhancement (LGE), qualitative and semi-quantitative stress perfusion (myocardial perfusion reserve index; MPRI) and strain imaging. Clinical, demographics, rejection score and CAV history were recorded and correlated with CMR parameters.

**Results:**

Mean age at transplant was 9.3 ± 5.5 years and median duration since transplant was 5.1 years (IQR 7.5 years). One patient had active rejection at the time of CMR, 11/26 (42%) had CAV 1 and 1/26 (4%) had CAV 2. Biventricular volumes were smaller and cardiac output higher in PHT vs. healthy controls. Global T1 (1053 ± 42 ms vs 986 ± 42 ms; p < 0.001) and ECV (26.5 ± 4.0% vs 24.0 ± 2.7%; p = 0.017) were higher in PHT compared to helathy controls. Significant relationships between changes in myocardial tissue structure and function were noted in PHT: increased T2 correlated with reduced LVEF (r = − 0.57, p = 0.005), reduced global circumferential strain (r = − 0.73, p < 0.001) and reduced global longitudinal strain (r = − 0.49, p = 0.03). In addition, significant relationships were noted between higher rejection score and global T1 (r = 0.38, p = 0.05), T2 (r = 0.39, p = 0.058) and ECV (r = 0.68, p < 0.001). The presence of even low-grade CAV was associated with higher global T1, global ECV and maximum segmental T2. No major side effects were noted with stress testing. MPRI was analyzed with good interobserver reliability and was lower in PHT compared to healthy controls (0.69 ± − 0.21 vs 0.94 ± 0.22; p < 0.001).

**Conclusion:**

In a PHT population with low incidence of rejection or high-grade CAV, CMR demonstrates important differences in myocardial structure, function and perfusion compared to age-matched healthy controls. Regadenoson stress perfusion CMR could be safely and reliably performed. Increasing T2 values were associated with worsening left ventricular function and increasing T1/ECV values were associated with rejection history and low-grade CAV. These findings warrant larger prospective studies to further define the role of CMR in PHT graft surveillance.

## Background

Pediatric heart transplantation (PHT) is the definitive treatment for end-stage heart disease associated with various pediatric and congenital cardiac diagnoses. Although improving over time, the long-term outcome from the most recent analysis of the International Society for Heart and Lung Transplantation’s (ISHLT) registry shows that overall survival 25 years post-PHT is 37% [1] with graft failure being the most common cause of death at all time points. This vulnerable population must undergo lifelong surveillance for development of comorbidities like coronary allograft vasculopathy (CAV) and rejection, two of the major causes of graft failure [2–4]. Although mortality has decreased over the last decade, those treated for rejection in the first-year post-transplant, and those with CAV have worse survival [2, 3].

Endomyocardial biopsy and invasive coronary angiography remain the gold standard for detection of acute rejection and CAV, respectively, but are invasive procedures with imperfect disease detection and several potential complications that multiply over repeated exposures, especially in younger patients [5–9]. Surveillance in PHT relies heavily on non-invasive imaging with echocardiography being the most commonly used modality in clinical practice. Some studies have shown that changes in echocardiography based myocardial deformation are associated with acute rejection and CAV [10–15] and potentially predictive of outcomes following heart transplantation [16], but these findings are subject to significant variability between patients, vendors and observers. Currently, no non-invasive test consistently and accurately diagnoses and/or predicts rejection and CAV [4].

Cardiovascular magnetic resonance imaging (CMR) has emerged as a tool for comprehensive assessment of myocardial structure and function. Compared to echocardiography, CMR more accurately represents ventricular volumes and function [17]. Similar to speckle tracking echocardiography, CMR-derived myocardial feature tracking analysis allows for reliable assessment of changes in left ventricular (LV) strain [18]. Late gadolinium enhancement (LGE) allows for assessment of myocardial scarring and other CMR tissue characterization techniques such as T2 and T1 mapping with calculation of extracellular volume fraction (ECV) allow for quantitative assessment of edema and myocardial fibrosis, respectively [19]. In addition, qualitative and quantitative myocardial perfusion assessment obtained via vasodilator stress regadenoson CMR perfusion imaging has been shown to be safe in adult heart transplant recipients, with similar side effect profile compared to patients with other indications [20]. There is emerging data reporting on the use of CMR in diagnosing graft dysfunction in the heart transplant population. These techniques have shown utility in diagnosis of acute rejection, CAV and clinical outcomes in adult patients [20–36] but data in the pediatric population are limited [37–43].

In this study, we aimed to (1) describe the safety and feasibility of performing vasodilator stress regadenoson CMR in the PHT population (2) characterize myocardial tissue, function and perfusion in a cohort of PHT and compare with healthy controls and (3) explore associations of CMR parameters with history of rejection or CAV.

## Methods

### Study cohort

We conducted a single-center retrospective cross-sectional case–control study that was IRB approved and HIPAA compliant. Informed consent was waived. PHT recipients who underwent a clinically ordered comprehensive stress perfusion structure–function CMR between May 2016 and January 2019 were identified. Eighteen historical institutional controls who had a normal structure–function CMR (without stress perfusion) for various indications including abnormal electrocardiogram (ECG) (n = 9), chest pain (n = 2), syncope (n = 2), family history of cardiomyopathy (n = 1), obesity (n = 1), suspected atrial mass (n = 1) and other systemic diseases (n = 2) were age and sex-matched to the PHT. Since there were no institutional healthy control patients who had undergone stress perfusion imaging, we identified a separate cohort of 16 non-transplant patients who underwent stress perfusion CMR with negative results performed for various indications where suspicion for microvascular dysfunction was minimal. Indications for CMR in this cohort were: post-coronary artery unroofing with chest pain (n = 4), single coronary artery (n = 2), history of arterial switch procedure (n = 1), failing Fontan (n = 1), remote history of Kawasaki disease with normal coronaries (n = 6), congenital coronary artery aneurysm (n = 1) and aortic stenosis with syncope (n = 1).

### Clinical data

Demographic and clinical data collection included transplant surgical history, number of rejection episodes and history of CAV per clinical history or cardiac catheterization report. A Rejection Score (RS) was devised to quantify the clinical rejection history by adding the total number of rejection episodes (acute cellular rejection ≥ 1B, any episode of antibody mediated rejection (AMR) based on endomyocardial biopsy (EMB) as per ISHLT criteria [44] or any episode of biopsy negative rejection that was treated clinically) in order to generate a score. In addition, CAV grading was performed by independent expert blinded review of coronary angiograms from the cardiac catheterization done closest to the time of the CMR by an adult interventional cardiologist with > 40 years of clinical experience. Angiograms were graded based on ISHLT CAV scale [45].

### CMR acquisition

**Fig. 1:**
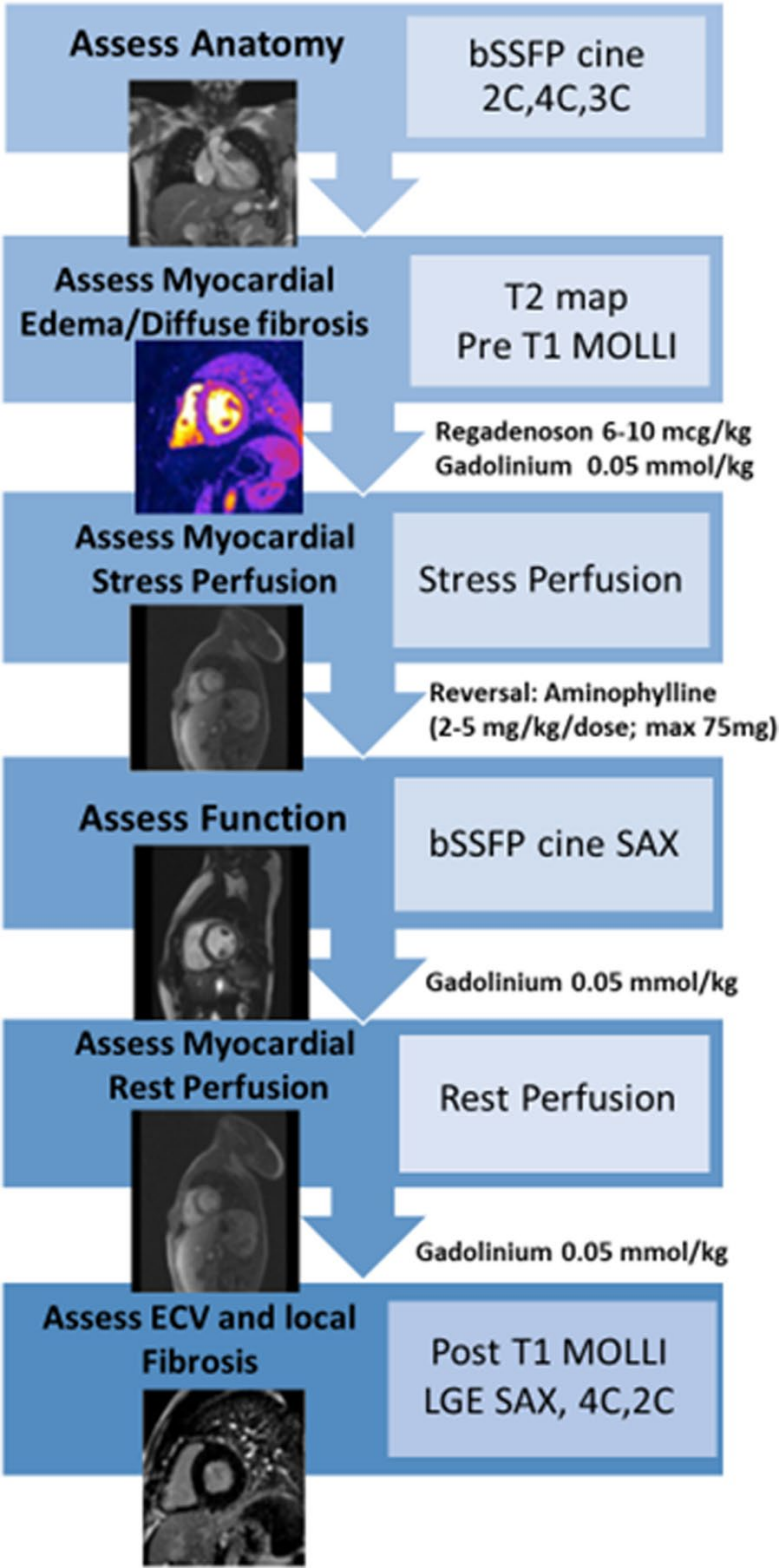
Schematic representation of the cardiovascular magnetic resonance (CMR) protocol for evaluation in pediatric heart transplantation (PHT) patients

CMR was performed on a 1.5 T scanner (Aera, Siemens Healthineers, Erlangen, Germany). A comprehensive protocol including structure and function assessment and stress perfusion testing was followed (Fig. 1) with the use of gadobutrol (Gadavist, Bayer HealthCare, Berlin, Germany), as the gadolinium-based contrast agent and regadenoson (Lexiscan; Astellas Pharm; Northbrook, Illinois, USA) as the pharmacologic stressor. Regadenoson is a selective cardiac A2A adenosine receptor agonist that vasodilates the coronary arteries thereby increasing myocardial blood flow.

Following image localizer scans, 2D cine balanced steady-state free-precession (bSSFP) images in the two-chamber, three-chamber and four-chamber planes were obtained in standard clinical fashion (TR = 3.0 ms TE = 1.26–1.3 ms; flip angle = 90°, slice thickness = 6 mm, in plane resolution = 1.0 × 1.0 mm^2^). T2 mapping was performed prior to contrast injection. Three T2-prepared bSSFP images with varying T2-prep times (0, 24, and 55 ms) were acquired in a breath-held fashion in three short axis orientations (base, mid, apex) through the LV (TR = 2.5 ms, TE = 1.1 ms, slice thickness = 8 mm, in-plane resolution 1.9 × 1.9 mm^2^). These levels were pre-selected by the technologist at the time of the scan: basal level defined at the mitral valve leaflet tips, apical level near the apex where the ventricular cavity was still visible at end-systole and mid ventricular level mid-way between those. Next, T1 mapping with a modified Look-Locker inversion recovery (MOLLI) sequence was used to measure native (pre-contrast) longitudinal relaxation T1 times of myocardium at the same three levels as well as within the blood pool. Pre-contrast T1 maps were obtained using a single-breath-hold, ECG-triggered, MOLLI sequence (TR = 2.6 ms, TE = 1.0 ms, slice thickness = 6 mm, in-plane resolution 0.7 × 0.7 mm^2^). Myocardial stress perfusion was then performed using regadenoson at a dose of 6–10 mcg/kg (up to a maximum dose of 400 mcg) [46] with close patient monitoring of symptoms and vital signs. First-pass contrast-enhanced images were acquired at 60–90 s at the same three short axis levels. Single-shot fast gradient echo, advanced motion corrected fast gradient echo sequences were acquired during stress after the intravenous administration of 0.05 mmol/kg of gadobutrol (TR = 2.5 ms TE = 1.1 ms, inversion time (TI) 150 ms, flip angle = 12°, slice thickness = 8 mm, inplane resolution = 2.8 × 2.8 mm^2^). Following image acquisition, a single dose of 75 mg of intravenous aminophylline was given to reverse the effects of regadenoson. Between stress and rest perfusion, ECG-gated 2-D Cine bSSFP imaging of the ventricles in short-axis from base to apex was obtained. Contrast-enhanced first pass perfusion imaging at rest was obtained following a second 0.05 mmol/kg dose of gadobutrol. Finally, a third dose of 0.05 mmol/kg dose of gadobutrol was injected with post-contrast T1 mapping. LGE was then obtained using segmented inversion-recovery sequences in three orientations: 4-chamber, 2-chamber and short axis from base to apex (TR = 2.8 ms, TE = 1.2 ms, slice thickness = 8 mm, flip angle 50°, inplane resolution = 1.4 × 1.4 mm^2^) around 20–30 min from the time of the initial contrast injection. For LGE sequences, the inversion time was selected using an inversion time scout scan to optimally null the normal myocardium.

### Patient preparation and monitoring

Patients were advised to refrain from the use of caffeine containing products for 24 h prior to the study. A baseline ECG was obtained prior to start of the exam. Heart rate and blood pressure were recorded at the start of the CMR and monitored during the test. A pediatric cardiologist, pediatric radiologist, a technologist and a nurse were present during induction of stress. Following administration of regadenoson, vitals were recorded every minute for 5 min, every 5 min for the next 20 min and then every 10–15 min until an hour from the time of stress.

Adverse events were documented and classified as major or minor. Major adverse events would result in need for termination of the study, and included arrhythmia, hypotension, atrioventricular block and bronchoconstriction. Minor adverse events would not typically result in termination of the test and included anxiety, chest pain, tingling in the limbs and gastrointestinal effects such as nausea and abdominal pain. Patients were monitored for an hour after the completion of the study prior to discharge.

### CMR post-processing

#### 2-D cine bSSFP and global function

Volumetric and functional data analysis from the 2D cine bSSFP short-axis stack was performed using dedicated software (Q Mass, version 7.2 Medis Medical Imaging Systems, Leiden, The Netherlands) by manual segmentation of the epicardial and endocardial borders. LV and right ventricular (RV) volumes, ejection fraction and cardiac output and LV mass were calculated.

#### T2 mapping

Endocardial and epicardial contours were traced on each T2 map excluding blood, epicardial fat or artifact. Images were motion corrected. An automated pixel-wise fit was used to generate a color map and analysis was done by drawing overlying regions of interest by dividing the myocardium into 16 segments as standardized by the American Heart Association (AHA) [47]. Segmental values were averaged to obtain the mean global T2 value. In addition, the maximum segmental T2 was recorded.

#### T1 mapping and ECV

Endocardial and epicardial contours were traced on each pre- and post-contrast T1 map to exclude blood, epicardial fat or artifact. Images were motion corrected. Similar to T2 mapping, color maps were generated, and analysis was done by drawing overlying regions of interest by dividing the myocardium into 16 segments as standardized by AHA. Extracellular volume fractio (ECV) was calculated for all 16 segments using the pre- and post-contrast maps and hematocrit using standard methodology [48]. Segmental values were averaged to obtain the mean global pre- and post-contrast T1 and ECV values. In addition, maximum segmental T1 and ECV values were recorded.

#### LGE analysis

For LGE analysis, we identified the presence or absence of focal myocardial fibrosis based on visual assessment as areas of relatively increased signal intensity following administration of gadolinium contrast. Myocardial fibrosis was noted only if LGE was seen in two orthogonal planes. LGE distribution patterns were classified as subepicardial, mid-myocardial, subendocardial or transmural and by AHA segment involved. This was confirmed by two experienced CMR readers at the time of the study interpretation.

#### CMR stress perfusion

Qualitative myocardial perfusion was assessed by two experienced CMR readers, one pediatric cardiologist and one pediatric radiologist, at the time of the clinical study interpretation and presence of perfusion defect determined by consensus review. If a deficit was present at stress but not at rest, it was described as an inducible, reversible perfusion deficit. If the defect was present both at rest and stress, and LGE was seen in the same myocardial region, it was described as fixed perfusion deficit. If the defect was present at rest and stress, and there was no LGE noted, it was considered an artifact. The presence of any regional wall motion abnormalities was used as an additional tool to aid in making the determination. Presence of dark rim artifacts were noted if present. Timing of hypointensity, symmetry of the lesion and comparison to resting perfusion were used to distinguish artifact from true perfusion defects. These were described as subendocardial or transmural (greater than 25% of the wall thickness) in pattern.

Semi-quantitative analysis by calculation of a myocardial perfusion reserve index (MPRI) was performed with the commercially available software (Medis Suite 3.0, Medis Medical Imaging Systems, Leiden, Netherlands). The software was used to obtain time signal intensity (TSI) curves for each AHA segment in the basal, mid and apical slices during stress and rest states. Maximum upslope was calculated as the maximum rate of signal intensity increase per unit time (au/s) and relative upslope was obtained by dividing the maximum upslope of the segment TSI curve with the maximum upslope of the myocardial blood pool TSI curve. The MPRI was then calculated as the ratio of the stress and rest relative upslopes for each segment and a global MPRI was calculated as the mean MPRI of all segments. A subset of PHT subjects (n = 9) were re-analyzed by a blinded second reviewer to assess for inter-observer differences.

#### Feature tracking

Feature tracking (FT) analysis was performed using dedicated post-processing software (QStrain, version 7.2; Medis Medical Imaging Systems). Standard 2-D cine bSSFP images in long and short axis which had 20 phases per cardiac cycle were used. Endocardial borders were manually drawn in end-diastole and end-systole. Four, three and two-chamber views were used to derive the peak global LV longitudinal strain (GLS) and peak systolic and diastolic strain rates. Three short-axis cine imaging slices (base, mid and apex) were used to derive peak global LV circumferential strain (GCS) and peak systolic and diastolic strain rates. The AHA 16 segment LV model was used to create a map of LV segmental systolic and diastolic strain and strain rate in order to register myocardial tissue and function data on the same anatomic map. A subset of PHT subjects (n = 5) were re-analyzed by a blinded second reviewer to assess for inter-observer differences in global longitudinal and circumferential strain.

### Statistical analysis

Data are reported as means ± standard deviations or median (interquartile range, IQR) as appropriate. Intraclass correlation coefficient was calculated to assess inter-observer variability in MPRI and FT strain in a subset of patients. To assess significant group differences, an unpaired t-test (for normally distributed data) or a Wilcoxon rank-sum test (for non-normally distributed data) was performed between cases and controls. Segmental differences in the CMR parametric data were displayed as AHA 16-segment maps. Additionally, Pearson’s correlation coefficients (r) were obtained to determine the relationships between clinical variables and CMR structure/function variables. Univariate and multivariable logistic regression model was used to determine association of CMR parameters with the rejection score. Significance was determined by p < 0.05.

## Results

Twenty-six PHT patients underwent 30 clinician-ordered CMRs during the study period (mean age 16.3 ± 3.1 years, 54% female). Individual patients were used as subjects with the most recent CMR used for evaluation. All patients underwent CMR around the time of their annual surveillance catheterization with a median of 28 days (IQR 42 days) between the procedures. All CMR exams in PHT and controls were performed without the use of anesthesia. Demographic differences are highlighted in Table 1. Mean age at transplant was 9.3 ± 5.5 years and the median duration since transplant was 5.1 years (IQR 7.5 years). Mean bypass and cross clamp times for PHT were 182 ± 53 and 176 ± 70 min respectively. The median RS was 5 (IQR 4). The age at last cardiac catheterization was 16.3 ± 3.1 years. Eleven of 26 PHT patients (42%) had CAV 1, one (4%) had CAV 2 and the remaining 14 (54%) had CAV 0. Only one patient was found to have clinically significant AMR at the time of the CMR.

**Table 1:** Demographics

	PHT (n = 26)	Healthy Control (n = 18)	p value
Age at CMR (years)	16.3 ± 3.1	16.3 ± 3.0	0.82
Sex (% female)	54%	50%	0.81
Height (cm)	164.0 ± 12.0	162.0 ± 9.0	0.55
Weight (kg)	62.4 ± 20.8	63.0 ± 15.0	0.89
BSA (m^2^)	1.7 ± 0.3	1.7 ± 0.2	0.93

*BSA* body surface area

Values reported as mean ± standard deviation unless specified

### Structure and function assessment by CMR in PHT recipients versus controls

**Fig. 2:**
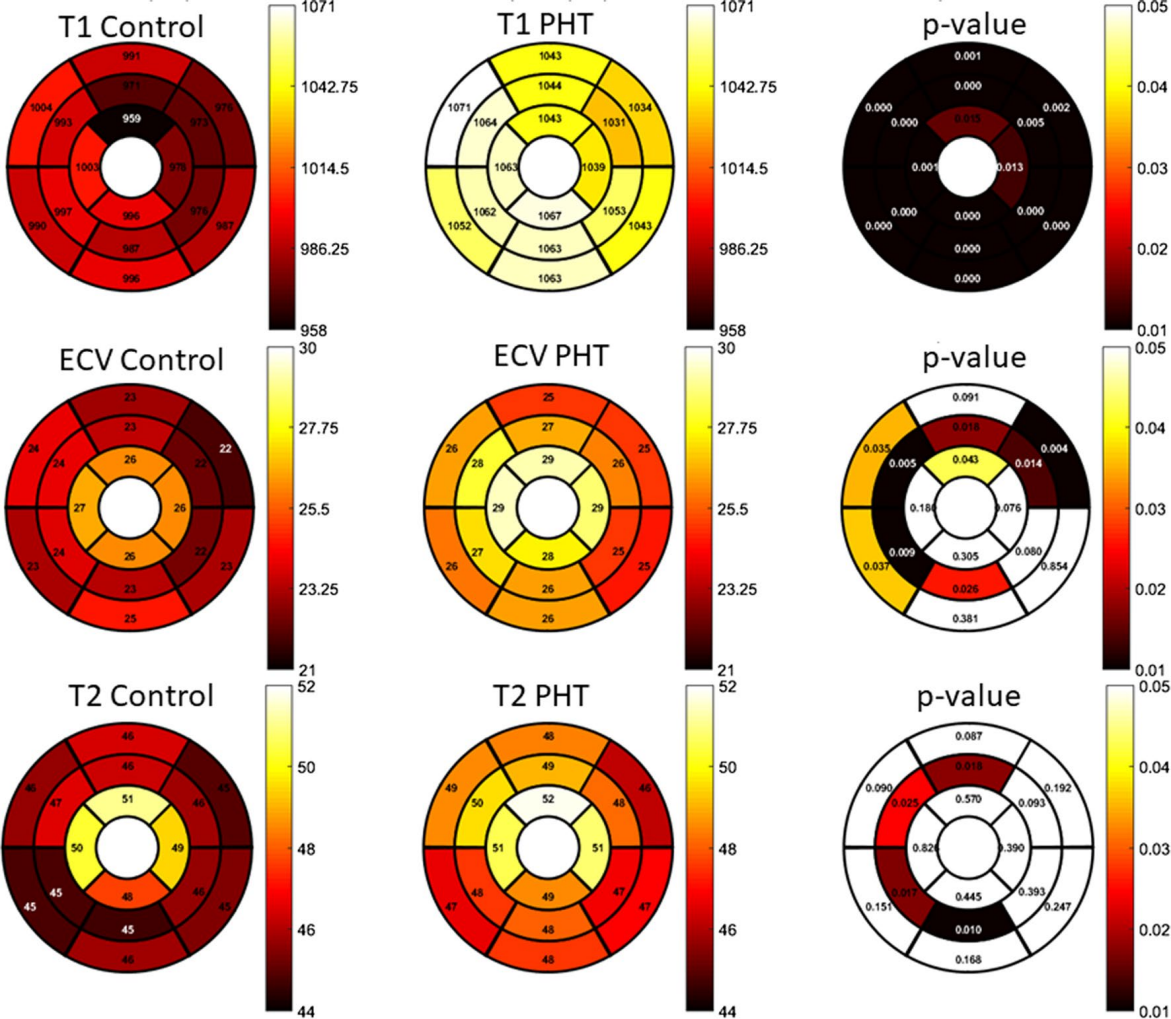
Segmental distribution (American Heart Assocaition (AHA) 16-segment bullseye model) of **a** T1 mapping **b** extracellular volume fraction (ECV) values and **c** T2 mapping. For each parametric variable, the first bullseye map shows the segmental values for controls, the second shows those values for PHT  and the third shows the p-value for differences between those variables in PHT versus controls in a segmental fashion. Scale for color grading is to the right of the maps

Ventricular volumes were smaller and cardiac output was higher in PHT compared to controls (Table 2). Baseline heart rate (prior to administration of regadenoson) was also higher in PHT. Global pre-contrast T1 and ECV were significantly higher and pre-contrast T1 was higher in all 16 AHA segments (Fig. 2) compared to controls. Nine of the 16 (56%) AHA segments (basal and mid-ventricular septum, basal and mid-ventricular anterolateral wall and the mid-ventricular and apical anterior walls of the LV) showed significantly elevated ECV as well. These segments corresponded mostly with left anterior descending coronary artery (LAD) territories with some overlap with right coronary artery (RCA) distribution. Global T2 values were not significantly different in PHT compared to controls, however 4/16 (25%) of the AHA segments (mid-ventricular septum, mid-anterior and mid-inferior walls) did show significantly elevated T2 in PHT. These segments corresponded to both LAD and RCA territories, similar to ECV. LGE was present in three (11.5%) PHT and none of the controls. There was no particular coronary distribution pattern, with LGE demonstrated in the mid-myocardial apical inferior wall (n = 2), inferior septal hinge point (n = 1) and subepicardial to mid-myocardial circumferential distribution from base to apex (n = 1).

**Table 2:** CMR variables

	PHT (n = 26)	Control (n = 18)	p value
Baseline heart rate (beats/min)	86 ± 9	70 ± 13	< 0.001
LVEDVI (ml/m^2^)	78.1 ± 13.6	91.8 ± 17.7	0.006
LVESVI (ml/m^2^)	32.7 ± 8.9	37.2 ± 7.5	0.09
LVSVI (ml/m^2^)	44.3 ± 8.5	54.5 ± 11.1	0.001
LVCI (l/min/m^2^)	4.5 ± 0.9	3.5 ± 1	0.003
LVEF (%)	58.4 ± 6.5	59.3 ± 2.7	0.58
LVmass index (g/m^2^)	46.2 ± 8.1	47.5 ± 13.7	0.97
RVEDVI (ml/m^2^)	76.1 ± 13.5	91.7 ± 17.7	0.002
RVESVI (ml/m^2^)	32.5 ± 9.3	41.8 ± 11	0.004
RVSVi (ml/m^2^)	43.5 ± 7.9	52.3 ± 0.7	0.004
RVCI (l/min/m^2^)	4.5 ± 1.0	3.4 ± 1	0.002
RVEF (%)	57.8 ± 7.5	55.8 ± 3.9	0.32
Pre-contrast global T1 (ms)	1053.3 ± 41.9	986.1 ± 41.9	< 0.001
Pre-contrast max segmental T1 (ms)	1120.1 ± 64.6	1054 ± 60.3	0.006
Post-contrast global T1 (ms)	444.1 ± 72.3	483.1 ± 55.0	0.008
Global ECV (%)^a^	26.5 ± 4.0	24.0 ± 2.7	0.017
Global T2 (ms)^b^	48.5 ± 3.9	46.7 ± 1.9	0.09
Maximum segmental T2 (ms)^b^	54.1 ± 4.8	52.9 ± 4.1	0.39

*LV* left ventricle, *RV* right ventricle, *EDVI* end-diastolic volume indexed, *ESVI* end-systolic volume indexed, *SVI* stroke volume indexed, *CI* cardiac index, *EF* ejection fraction

Values reported as mean ± standard deviation unless specified

^a^Available data on n = 24

^b^Available data on n = 21. Indexing done to BSA

**Fig. 3:**
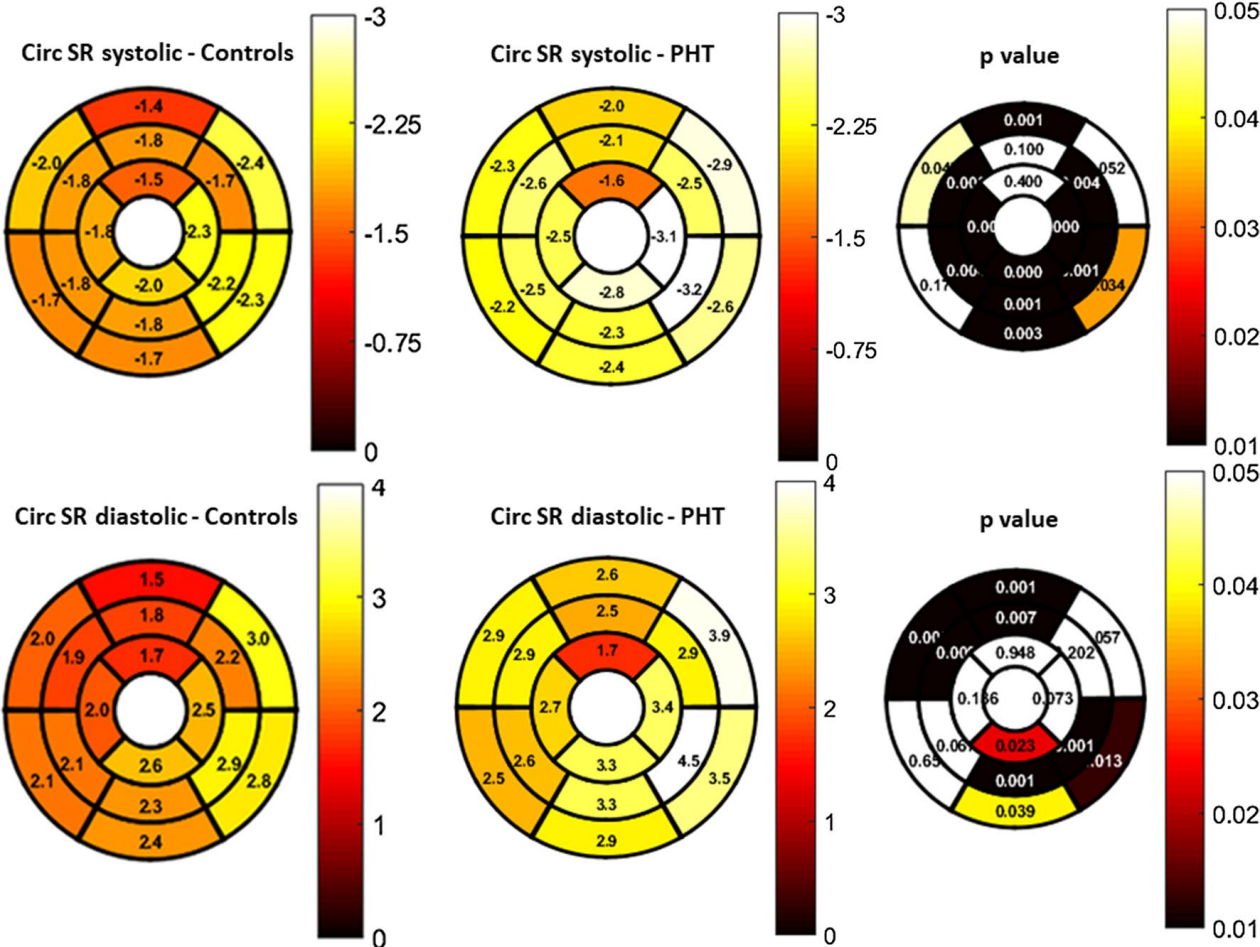
Segmental distribution (AHA 16-segment bullseye model) and p-values for differences between  healthy controls and PHT in systolic and diastolic global circumferential strain rate (Circ SR). Scale for color grading is to the right of the map

**Fig. 4:**
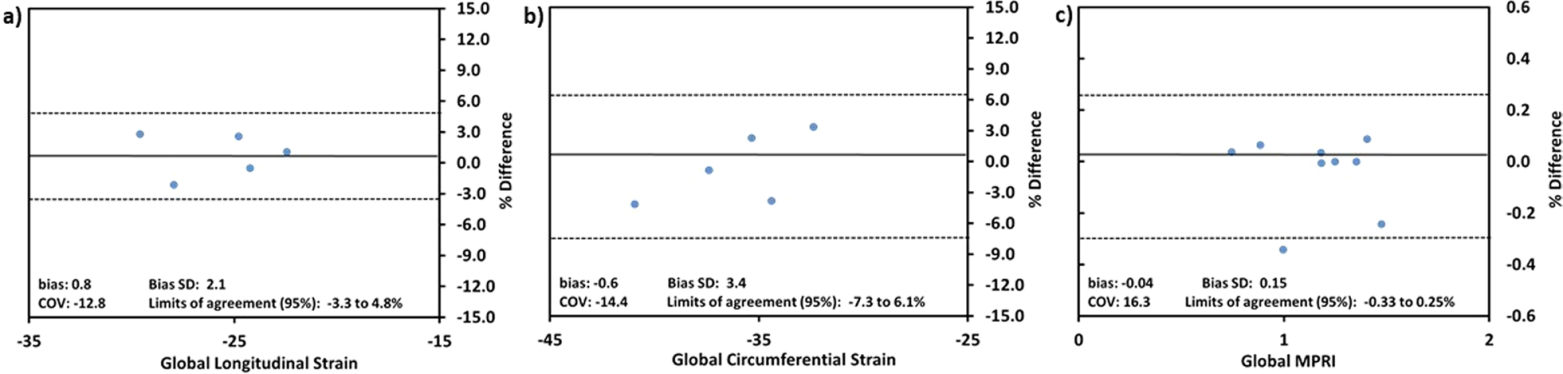
Bland–Altman plots showed agreement and absence of significant bias between two blinded observers measuring **a** global longitudinal strain **b** global circumferential strain **c** global myocardial perfusion reserve index (MPRI)

GLS rate and GCS rate were higher in this cohort of PHT (Table 3). All mid-ventricular and apical segments and most of the basal segments had significantly higher systolic circumferential strain rate (Fig. 3). A Bland–Altman analysis between two blinded reviewers showed agreement with no significant bias in GLS (bias: 0.8 ± 2.1, 95% LOA − 3.3 to 4.8%; p = 0.45) and GCS measurement (Bias: − 0.6 ± 3.4, 95% LOA − 7.3 to 6.1%; p = 0.71) (Fig. 4a, b).

**Table 3:** Strain by CMR feature tracking

Variables	PHT (n = 26)	Control (n = 18)	p value
Global circumferential strain (%)	− 35.06 ± 5.1	− 32.87 ± 2.1	0.09
Global longitudinal strain (%)	− 25.68 ± 4.5	− 25.12 ± 2.3	0.63
Global circumferential strain rate (/sec) in systole	− 2.48 ± 0.5	− 1.87 ± 0.3	< 0.001
Global circumferential strain rate (/sec) in diastole	2.98 ± 0.6	2.29 ± 0.4	< 0.001
Global longitudinal strain rate (/sec) in systole	− 1.63 ± 0.3	− 1.33 ± 0.2	0.003
Global longitudinal strain rate (/sec) in diastole	1.96 ± 0.4	1.63 ± 0.5	0.03

Values reported as mean ± standard deviation

### Stress perfusion analysis

Stress perfusion was reliably performed in 24/26 (92.3%) CMR exams. Deviation from standard protocol with exclusion of stress perfusion testing occurred because of the clinical situation (1 with active rejection) or patient factors (1 for limited scan due to difficulty lying in scanner). There were no major adverse reactions and 1/24 (4.1%) PHT reported a minor adverse reaction with nausea and gastrointestinal discomfort. Two of 16 (12.5%) perfusion controls had minor adverse reactions including limb numbness and chest discomfort that self-resolved. Qualitative inducible subendocardial defects were noted in 3/12 (25%) of PHT with known CAV: 1/1 (100%) with CAV2 and 2/11 (18%) with CAV1. These qualitative perfusion defects overlapped the CAV coronary territory in 3/3 (100%) PHT with positive findings. Compared to the control group, PHT were found to have a significantly lower global MPRI (PHT mean: 0.69 ± − 0.21; PHT control mean: 0.94 ± 0.22; p < 0.001) with no significant differences between CAV 0 versus CAV 1. MPRI did not correlate with ischemic time during heart transplant surgery. A Bland–Altman analysis between two blinded reviewers showed agreement with no significant bias in MPRI measurement (Bias: − 0.04 ± 0.15, 95% LOA − 0.33 to 0.25%; p = 0.44) (Fig. 4c).

### Structure–function relationships

**Fig. 5:**
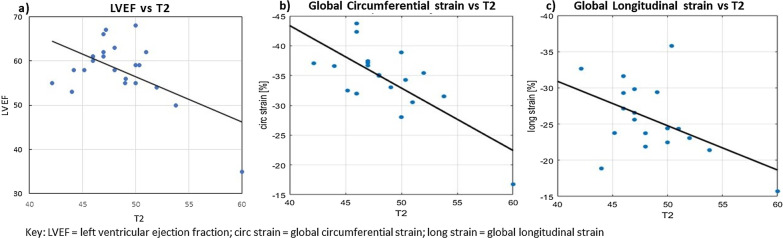
Correlation plot between **a** left ventricular ejection fraction (LVEF) (in %) versus T2 values (in ms); **b** GCS (in %) versus T2 values (in ms); **c** GLS (in %) versus T2 values (in ms)

There were significant correlations between tissue structure and cardiac function noted in PHT. Increased global myocardial T2 value was significantly associated with lower LVEF (r = − 0.57, p = 0.005), reduced GCS (r = − 0.73, p < 0.001) and lower GLS (r = − 0.49, p = 0.03) (Fig. 5). In addition, there were significant relationships between tissue parameters: T2 with ECV (r = 0.68, p < 0.001), and T2 with T1 (r = 0.68, p < 0.001).

### Clinical correlations with CMR

With increasing years since transplant, there was a significant increase in the global pre-contrast T1 values (r = 0.45, p = 0.03) and ECV values (r = 0.46, p = 0.02) in PHT recipients. There was higher GCS noted in those who were further out in years post-transplant (r = 0.45, p = 0.025). No correlations were noted between age and T2, T1 and ECV or MPRI.

**Fig. 6:**
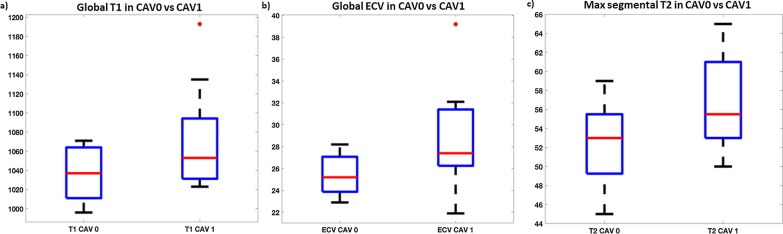
Box and whisker plots showing significant differences (p < 0.05) between coronary allograph vasculopathy (CAV) 0 and CAV1 in **a** global T1 values (in ms) **b** global ECV values (in %) and **c** maximum segmental T2 values (in ms). All differences shown are significant with red dots representing outliers

There were modest correlations between higher rejection scores and elevated global T1 (r = 0.38, p = 0.05), T2 (r = 0.39, p = 0.058) and ECV (r = 0.68, p < 0.001). Significant structural differences were also noted between CAV 0 versus CAV 1: CAV 1 showed higher global T1: (1037 ± 28 vs 1072 ± 53 ms; p = 0.04), higher global ECV (25.4 ± 1.9 vs 28.9 ± 4.6% p = 0.02) and higher maximum segmental T2 (52.3 ± 4.0 vs 56.5 ± 4.8; p = 0.04) (Fig. 6).

## Discussion

In recent years, CMR has shown substantial promise for detection of graft dysfunction in adult heart transplant recipients [21, 23, 24, 28, 37, 49–53], however, data are quite limited in PHT [37, 38, 40, 43, 54, 55]. Our study demonstrates the safety and feasibility of comprehensive CMR with regadenoson stress perfusion for graft surveillance in PHT. Compared to healthy controls, we found differences in biventricular volumes, LGE, global and segmental myocardial T1, ECV and T2 values, and MPRI. More importantly, we found significant relationships between CMR parameters and graft function which may indicate disease progression. Increased T2 values were associated with reduced systolic function as assessed by LVEF and GCS and GLS. Increased T1/ECV and T2 values were associated with clinical rejection and CAV history, with significant differences between patients with early (CAV 1) and no (CAV 0) allograft vasculopathy. Additionally, we noted that regadenoson was safe for use in stress imaging in PHT, with no major and infrequent minor adverse effects. While qualitative stress perfusion imaging identified inducible subendocardial defects in only 25% of patients with early-stage CAV, overall PHT patients with CAV 0–2 were found to have a significantly lower global MPRI compared to controls. These findings may be helpful in identifying early or progressive graft dysfunction due to the cumulative burden of CAV and rejection and could potentially help refine the timing of or need for more invasive procedures. Further investigations are warranted.

### CMR tissue characterization in PHT

As has been shown in previous adult studies [51], we found that myocardial native T1 and ECV were higher even in our relatively healthy PHT cohort. These differences were noted at the segmental level with elevated native T1 values in PHT in all AHA segments and elevated ECV in greater than 50% of the segments, in no specific coronary distribution pattern. Additionally, in this cross-sectional cohort we noted that with increasing duration since transplant, the T1 and ECV values rose, suggesting ongoing myocardial remodeling with time that may reflect progressive chronic graft failure. While global T2 values were not different between PHT and healthy controls, segmental differences were noted.

Many adult studies have shown that elevated native T1, T2 and ECV may be markers of acute rejection and CAV [21, 23–26, 28, 49]. One recent pediatric study showed that there may be trends in T2 that indicate allograft rejection [43]. In our cohort, only one subject had acute rejection at the time of CMR and that patient showed significant abnormalities in all three parametric variables (T1 = 1193 ms, ECV = 32.1% and T2 = 60 ms), which corroborates studies in which T2 signal has been indicated as a potential marker of acute rejection [23, 24, 26, 43]. In addition, T2 and ECV have shown to be independently associated with cardiac events (cardiac death, myocardial infarction, coronary revascularization, and heart failure hospitalization) and noncardiac death or hospitalization in adult patients [32]. We did not have longitudinal follow up of our PHT recipients yet and suggest that more studies with CMR performed at the time of acute rejection and significant CAV diagnosis are needed.

A small subset of our PHT cohort (11.5%) showed positive LGE. Studies in adult transplant recipients demonstrate that presence and severity of LGE predict long-term outcomes such as mortality and major adverse cardiac events [56, 57], and are more common with higher CAV grades [58], however, the role of LGE imaging in PHT surveillance is not established. In a small pediatric study [39], prognostic value of LGE in determining cellular rejection was not seen, although the study was likely underpowered to detect the outcome. Similar to our cohort, LGE is known to be fairly common in adult heart transplant recipients at the time of their first CMR [31] and appears to be relatively stable over time.

### CMR stress perfusion in PHT

CMR stress perfusion testing was reliably and safely performed in our study. Only one PHT in our cohort had a minor adverse event and no serious adverse events were noted. This low incidence is similar to that reported in other pediatric cohorts [46]. Three of 12 PHT with known CAV showed abnormalities on stress perfusion testing, suggesting that qualitative CMR perfusion assessment has a high positive predictive value (100%) but a low negative predictive value (61%) for detection of early CAV. In other words, qualitative perfusion assessment with its associated technical challenges in the pediatric population may not be an ideal rule in or rule out test for CAV detection. Given the diffuse and heterogenous nature of CAV, semi-quantitative MPRI and GLS have been described as promising potential markers of CAV in late-stage heart transplantation [34]. In our cohort, compared to controls, MPRI was decreased in PHT even without CAV suggesting that perfusion reserve is likely abnormal in most PHT recipients. These differences were noted despite the lack of a normal control cohort as described above. MPRI was not different between angiographically determined CAV 0 and CAV 1, however, this could be due to the relatively subjective nature of angiographic interpretation of early CAV and/or the absence of higher-grade CAV subjects in our cohort.

The safety profile, clinical findings and good reproducibility of MPRI measurements shown across observers, should motivate further studies to better quantify myocardial perfusion as an indicator of CAV in PHT.

### CMR functional assessment in PHT

Despite similar body sizes between PHT and age-matched controls, ventricular volumes in PHT were smaller even though cardiac output was overall higher. This has been previously described in pediatric cohorts [59, 60] in which a wide range of size mismatch between donors and recipients is necessary and acceptable [61]. These observed differences may be a consequence of initial size mismatching and chronic adaptive or maladaptive remodeling that occurs over time.

Echocardiography-derived strain and CMR derived myocardial velocities are often abnormal during an acute rejection episode or with cumulative history of repetitive rejections [11–13, 28, 37, 37, 40, 41, 62, 63]. Miller et al. [30] have reported an association of reduced GCS by CMR tagging with clinically significant ACR (grade 2 R or higher) in adult heart transplant recipients. Grotenhuis et al. [40] showed similar results in a pediatric cohort. Controversy exists, however, regarding which strain dimension is affected with rejection, with several echocardiography-based studies showing a reduction in GLS with preservation of GCS. Several studies have shown abnormal contractility following heart transplantation even in the absence of significant rejection [64, 65] and altered systolic deformation patterns in healthy transplanted hearts compared to native hearts [52]. LV echocardiography-derived strain parameters have also been shown to change over time in healthy PHT recipients [55]. Overall, patterns of strain are not well understood in those with acute or chronic rejection or in those with healthy transplanted hearts.

In our cohort, we noted that while GLS and GCS were not significantly different between PHT and healthy controls, there were significant differences in strain rate. We also observed higher GCS in PHT further from transplant. These differences could be due to a variety of factors including differences in age, heart rate, cardiac size, donor-recipient size mismatch or loading conditions. Importantly, the mechanistic and clinical implications are unclear given the cross-sectional nature of our study design and prospective, longitudinal studies could answer some of these questions and clarify significance.

## Limitations

This was a retrospective chart review with limitations inherent to this type of a study design. We had a small sample size with only 26 PHT and 18 age-matched healthy controls, which may have limited the strength of associations between clinical and CMR variables. In addition, true controls were not available for myocardial stress perfusion comparisons and therefore, we selected non-PHT subjects with other diagnoses who had undergone stress perfusion CMR with negative results and had minimal suspicion for microvascular dysfunction. Complete CMR protocol was not performed in all patients, leading to missing T2 data in five PHT, ECV data in one and MPRI in two patients. PHT recipients presented for their CMR following variable time since heart transplantation which somewhat limited evaluation of temporal relationships between clinical and CMR variables.

## Conclusions

In a PHT population with low incidence of CAV and rejection, CMR demonstrates important differences in myocardial structure, function and perfusion compared to age-matched controls. Increasing T2 values were associated with worsening LV function and increasing T1/ECV and T2 values were associated with rejection burden and low-grade CAV. These myocardial structure–function relationships may indicate early graft dysfunction. Larger, prospective studies are warranted to define the role of CMR in PHT graft surveillance.

## Data Availability

The data generated or analyzed during the current study are available from the corresponding author on reasonable request.

## Abbreviations

AHA: American Heart Association
AMR: Antibody mediated rejection
BSA: Body surface area
bSSFP: Balanced steady-state free-precession
CAV: Coronary allograft vasculopathy
CMR: Cardiovascular magnetic resonance
ECG: Electrocardiogram
EMB: Endomyocardial biopsy
ECV: Extracellular volume fraction
FT: Feature tracking
GCS: Global circumferential strain
GLS: Global longitudinal strain
IQR: Inter-quartile range
ISHLT: International Society of Heart and Lung Transplantation
LAD: Left anterior descending coronary artery
LGE: Late gadolinium enhancement
LOA: Limits of agreement
LV: Left ventricle/left ventricular
LVCI: Left ventricular cardiac index
LVEDVI: Left ventricular end-diastolic volume index
LVEF: Left ventricular ejection fraction
LVESVI: Left ventricular stroke volume index
MOLLI: Modified Look-Locker inversion recovery
MPRI: Myocardial perfusion reserve index
PHT: Pediatric heart transplantation
RCA: Right coronary artery
RS: Rejection score
RV: Right ventricle/right ventricular
RVCI: Right ventricular cardiac index
RVEDVI: Right ventricular end-diastolic volume index
RVEF: Right ventricular ejection fraction
RVESVI: Right ventricular stroke volume index
TE: Echo time
TR: Repetition time
TSI: Time signal intensity
